# Trade Strikes

**Published:** 1869-10

**Authors:** 


### TRADE STRIKES.

One of the mischievous tendencies which the idleness incident to men who cease work in order to compel higher wages is the congregating about drinking-places, the cultivation of angry feelings against employers, against law, and against right.

All workmen admit that this is a free country, as free to employers as to themselves; suppose these employers were to rise in a body and strike for lower wages, or even to close their establishments, what then? Having means ahead and capital to embark in other avocations, they could well afford to do nothing or change their calling, so that they neither need be idle, nor live on the hard earned contributions of others.

No man of correct feelings can look at a locomotive engineer, a mason, a foundry man, a stoker or a hod-carrier without a certain degree of respect and admiration that they follow those laborious and grimy occupations through summer's heat and winter's cold, day in and day out, from one year's end to another, as a means of making a comfortable and honest living for themselves and dependent families, rather than resort to modes employed by better dressed and better educated men, as gaming, and fraud, and robbery, and downright theft. The truth is, that men and women who earn a living by day labor, are nothing short of moral heroes, and are the substantial supports of good society and of good government, and as such merit the regards and the respect of all the good, and it is sure that the thinking portion of them will regard with special attention the following considerations. In reference to all workmen's strikes, the first worst effects fall on themselves, because on the instant they not only cease to earn, but commence to use what they have earned, and certain destitution is in the future. Another important consideration is, that just in proportion as they succeed in getting higher wages they increase their own burdens and those of their own class in life and the greater multitude of those who are below them socially and pecuniarily, for in any department of trade, the more it costs to produce an article, the more money must be demanded for that article, for it cannot be expected that men will embark in manufacture and sell their wares for the bare cost of production, or even simple interest, because if they are to get no more than the interest of their money, it would be easier to loan it out on bond and mortgage and spend their time in enjoying themselves, instead of working for nothing; and that is the virtual aim of the striker, to get more money for his time, and have his employer work for less. But a nobler course than that of strikes, and strifes, and angry contentions is simply this: if a workman cannot make a comfortable living by his present occupation, let him change his calling. This is the course pursued by men of capital, and men of intelligence and enterprise without capital; if what they are at does not pay, they seek something that will pay, and keep on seeking with patient courage and an independent determination until they find a calling which is encouragingly remunerative; they don't go around among their relations and friends, and business acquaintances, levying contributions to help them continue in waiting for higher wages and a better remuneration. As a suggestion to workmen who are dissatisfied with the compensation which they are receiving for their labor, we will simply narrate

### The Story of a Man.

On the steamer passing last summer from Shediac to Prince Edward Island, a person among the deck passengers, from his manly build and proportions, attracted our attention. He was plainly but substantially dressed. There was about him a certain air of quiet confidence and self appreciation, which, with his evident desire to be alone, or at least as remote from the more common of those on his part of the ship, induced us to engage in conversation with him. He used good language and spoke in a quiet tone, such as to impress the hearer with the truthfulness of what he was saying. After drawing him out we let him talk āway without interruption, which was in substance as follows: A year ago I had a wife and young child and a little money, but times were dull; I could not get work, and our little pile was daily growing less. Wife and I talked it over and it was determined that I should leave her and our little baby and go to Minnesota, which I did at once; and finding a farm of eighty acres for sale, with very few improvements, at a price a little above what government asked, I purchased and paid for it, and then entered another tract of eighty acres; I went to work, kept at it all winter, and in the spring put in some corn and vegetables and a little wheat; the season proved favorable and my crops being in a favorable state of forwardness, I concluded to leave them in the hands of a trusty man, and am now on my way home, and expect in ten days to see my wife and child after a years' absence; and about the first week in September I hope that we shall all be in our own home; and as I have not been sick an hour, and they have been well all the time, I trust we shall have brighter days; to be away from them a whole year was a bitter, bitter pill; but now I have a home for life, all paid for, and have great reason to be thankful. Let our honest high-minded workmen, who are not satisfied with their wages, think of this and of the further fact, that there are millions of acres of cultivatable land in the West belonging to the government, which says to them, make your selection, go to work and pay for it in the future; and think of the happiness of having a farm and home of your own, where all the profits of your labor will go into your own pocket, with the promise of glorious good health thrown in.

